# Health Management Practices, Antimicrobial Use and the Adoption of Alternatives Among Nigerian Fish Farmers: A Mixed‐Methods Study

**DOI:** 10.1155/vmi/3100926

**Published:** 2026-10-28

**Authors:** Ismail Ayoade Odetokun, Jadesola Juliana Ajao, Afisu Basiru, Rodiat Olabisi Omotoso, Mutiat Adenike Adetona, Wasiu Adeyemi Jimoh, Somrat Adeola Abdulsalami, Abdulfatai Aremu, Mutiat Olaitan Mohammed, Samira Sarih, Enric Gisbert, Maria Dolores Furones Nozal, Elena Garcia Fruitós, Anna Aris, Silvia Torrecillas, Mahmoud Eltholth

**Affiliations:** ^1^ Department of Veterinary Public Health and Preventive Medicine University of Ilorin Ilorin 240003 Nigeria unilorin.edu.ng; ^2^ Department of Health Studies Royal Holloway University of London Egham TW20 0EX UK; ^3^ Department of Veterinary Physiology and Biochemistry University of Ilorin Ilorin 240003 Nigeria unilorin.edu.ng; ^4^ Department of Statistics University of Ilorin Ilorin 240003 Nigeria unilorin.edu.ng; ^5^ Department of Aquaculture and Fisheries University of Ilorin Ilorin 240003 Nigeria unilorin.edu.ng; ^6^ Department of Fisheries, Aquaculture and Wildlife University of Abuja Abuja Nigeria uniabuja.edu.ng; ^7^ Department of Veterinary Pharmacology and Toxicology University of Ilorin Ilorin 240003 Nigeria unilorin.edu.ng; ^8^ Department of Biological Sciences Summit University Offa Nigeria; ^9^ Institut de Recerca i Tecnología Agroalimentaries (IRTA) Aquaculture Programme Torre Marimon Caldes de Montbui 08140 Catalonia Spain; ^10^ Hygiene and Preventive Medicine Department Faculty of Veterinary Medicine Kafrelsheikh University Kafr El-sheikh Egypt kfs.edu.eg

**Keywords:** antibiotics, antimicrobial alternatives, catfish, diseases, management, Nigeria, prevention, tilapia

## Abstract

This study assessed the associations between production practices and disease outbreaks on Nigerian fish farms while identifying factors that shaped farmers’ choices between antimicrobials and non‐antimicrobial health management interventions (NAHIs), including herbs and other commonly used interventions such as charcoal, formalin and salt. A mixed‐methods approach was employed, comprising questionnaire surveys (*n* = 330) and focus group discussions. Results indicated that only 12% of farms stocked their fish at the recommended stocking density (7 fish/m^2^) or below. Most farms (66.1%) shared water sources with neighbouring farms. Regarding health management expenses, the majority of farms (62%) reported spending on medication during the last production cycle, while fewer farms reported spending on biosecurity (20%) and water quality testing (15.5%). Most farms (63.3%) had survival rates of > 75% during the last production cycle. Less than half (27%) reported experiencing a disease outbreak over the last year. Some associations between production practices and survival rates were identified in Fisher’s exact test and Pearson’s chi‐square analyses, but none remained independently associated in the multivariate logistic regression model (*p* > 0.99). However, farmers who spent on biosecurity were less likely to have spent on medications than farmers who did not (OR: 0.29, 95% CI: 0.14–0.56, *p* : 0.001). Findings from the focus groups indicated that antimicrobials, primarily antibiotics, were utilised in combating severe disease outbreaks. Antimicrobials were frequently misused, with self‐prescribed medications administered before veterinary consultation or laboratory confirmation of disease aetiology. Some farmers reported reducing their antimicrobial usage after learning about antimicrobial resistance. Farmers also reported using a variety of NAHIs, primarily herbs, in health management. Antibiotics were considered more effective than NAHIs, with some farmers reporting adopting them for severe outbreaks. Adoption of NAHIs was influenced by farmers’ awareness and cost‐affordability, while uncertainty about correct dosage formulations prevented greater adoption. We recommend increasing awareness about preventive management and safe, regulated NAHIs for Nigerian fish farmers. Studies can also be embarked on to explore the efficacy potential of commonly used NAHIs. In addition, subsidised veterinary care could improve farmers’ uptake of these services.

**Highlights**
• Farm health management costs are largely directed towards treatment rather than prevention.
• Farmers’ awareness levels and consideration of cost‐affordability drive their use of non‐antimicrobial health interventions.
• Antibiotics are considered by farmers to be more effective than herbs.
• Farmers reported a reduction in antimicrobial use following awareness of AMR risks.

## 1. Introduction

Aquaculture is one of the world’s fastest‐growing food production industries, accounting for an estimated 53% of total global aquatic production [1]. Millions of people worldwide rely on farmed fish for animal‐based protein, and this sector provides income and employment opportunities to innumerable communities at the margins of national development priorities [2]. Nigeria is the second‐largest fish‐producing country in Africa, with an annual output of 261,621 tonnes valued at USD 600.7 million in 2020 [1]. The African catfish (*Clarias gariepinus*) is the most dominantly produced fish species in the Nigerian aquaculture industry, while tilapia (*Oreochromis niloticus*) is the second‐most commonly reared species, with a production of 69,579 tonnes in 2018 [1, 3]. As Nigeria’s population expands, there is further demand for affordable animal‐source protein. This has driven the intensification of aquaculture, leading to the increased production of catfish and tilapia and leading to improper production practices. These practices ultimately lead to water pollution, fish stress and disease [3, 4].

Disease outbreaks significantly hinder the economic viability of aquaculture by reducing productivity, increasing mortality rates, and raising production costs [5]. Globally, aquaculture disease outbreaks lead to an estimated loss of USD 1.05–9.58 billion every year [6]. In Nigeria, each catfish farm has been estimated to lose USD 192.79 to USD 2056.38 per production cycle to disease, a significant amount for the thousands of small‐scale farmers who dominate the Nigerian aquaculture industry [7]. Infectious agents, including *Aeromonas hydrophila*, *Pseudomonas septicemia* and *Streptococcus* spp., have been implicated in disease outbreaks in fish [8]. To curtail disease outbreaks, farmers often resort to using antimicrobials, primarily antibiotics. Antibiotics are also misused in aquaculture for prophylactic and growth‐promotive purposes [9]. Alhaji et al. [10] reported that 95% of fish farmers in Northern Nigeria self‐prescribed antimicrobials, and 53% administered antibiotics to their ponds weekly regardless of disease status.

Widespread misuse of antibiotics is a key driver of antimicrobial resistance (AMR) in aquaculture, which manifests as an emergence of antimicrobial resistance genes (ARGs) in fish products and an increase in antibiotic residues above permissible levels for human consumption [11, 12]. This highlights the One Health implications of AMR in aquaculture, as well as the need to discover transdisciplinary solutions that protect both human and fish health [10, 13]. One potential solution is the discovery and promotion of non‐antimicrobial health interventions (NAHIs), which are regarded as alternatives to antimicrobials, for use in fish farming [10, 14]. These are agents that can be used to prevent and treat diseases without the use of conventional antibiotics. They include peptides, probiotics, herbs, bacteriophages, immunostimulants and vaccines [14–16]. Practices such as biosecurity and proper management are also important antimicrobial alternatives, as they improve fish health and reduce the possibility of disease outbreaks [17].

While preventive practices and NAHIs offer significant potential for reducing antimicrobial use (AMU), various reasons prevent their widescale adoption in aquaculture production. Many NAHIs, including herbal plants, remain understudied, with generally limited information about their effectiveness across different aquaculture systems [14, 15]. Preventive products supported by strong research and development efforts, such as vaccines, still remain underutilised in major fish‐producing nations of the world [14]. A potential reason for this is fish farmers’ personal perceptions. For instance, a study in Vietnam reported that some fish farmers had concerns about the efficacy of vaccines and were less likely to be open to using them in the future [18]. Evidence‐supported practices of biosecurity and good management have also been observed to be inconsistently implemented on Nigerian farms, partly due to farmers’ lack of awareness[3, 7, 10].

It is important to gain insight into the health management practices of Nigerian farmers, as well as the factors influencing their decisions between antimicrobials and NAHIs. It is equally essential to study the production practices that underpin Nigerian aquaculture production, as these may reveal risk factors for disease outbreaks and subsequently the use of antimicrobials and NAHIs [3, 4]. Thus, this mixed‐methods study attempts to contribute towards the existing research gap by (i) characterising the aquaculture production system in Nigeria and the costs of operation; (ii) examining how production practices and cost allocation decisions might influence disease outbreaks and health management and vice versa; and (iii) identifying how farmers’ personal perceptions shape the use of antimicrobials and NAHIs.

## 2. Materials and Methods

### 2.1. Study Area and Design

This was a sequential mixed‐methods study incorporating exploratory and explanatory phases. The study was conducted in commercial aquaculture farms across two states in Nigeria: Kwara (location: 8° 30′N and longitude 5° 00′E) and Lagos (location: latitude 6° 27′14.65′N and longitude 3° 23′40.81′E). A map of the study areas is attached as Figure 1. These two states were selected as their production represents a significant portion of Nigeria’s aquaculture [19, 20]. The study consisted of three parts. First, an initial pilot visit to each state provided contextual information regarding farming‐dense regions. During this visit, a preliminary focus group discussion (FGD) was organised in Lagos State to provide contextual understanding regarding farmers’ health management practices. Afterwards, a quantitative survey was conducted in selected farms across both states with the aid of a semistructured questionnaire. Finally, another FGD was organised in Kwara State to further explain results obtained from the questionnaire survey. The results from the initial FGD influenced the design of the questionnaire, while findings from the questionnaire influenced the questions asked during the second FGD.

**FIGURE 1:**
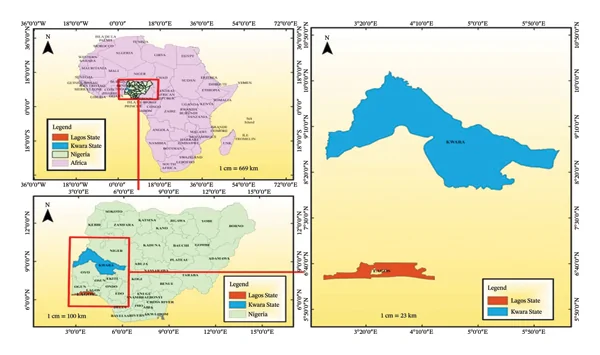
Geographical location of the study participants (Lagos and Kwara States) highlighted in red and blue.

### 2.2. Sampling Procedure

The target population for the questionnaire survey was fish farming stakeholders in both states, including farm owners, managers and workers. Within Lagos and Kwara, respondents were recruited from individual farms in farming‐dense regions, called ‘farming villages’, across both states. Lagos farming villages included Akute, Badagry, Epe and Ikorodu. Kwara farming villages included Aliara, Egbejila (Phases 1–4), Kilanko and Offa. Farming villages were largely comprised of dozens of catfish, tilapia, or mixed farms, rendering all establishments eligible for inclusion in this study. Individual farms were selected using simple random sampling. The sample size was determined using OpenEpi (v2.3.1). The calculation assumed an estimated 50% expected frequency, 95% confidence interval, 6% desired absolute precision, a design effect of 1.0 and an unknown population size. Allowing a 15% nonresponse rate, this resulted in a calculated sample size of a minimum of 315 respondents. A total of 330 respondents were eventually recruited to mitigate potential issues that could arise from invalid responses. Each farm was visited by a lead researcher and 2–3 research assistants. The farm manager or owner was approached first, and the reason for the visit was explained. If an approval was granted, the lead researcher selected one respondent per farm, based on their willingness to participate and knowledge of the farm’s management and production practices. The latter was also confirmed by the farm manager.

For the FGDs, 16 participants were recruited across Kwara and Lagos. Priority was given towards ensuring demographic diversity (age, sex and farm size) and representation of key stakeholder groups (fish farmers, feed millers, veterinarians and laboratory scientists). A list of FGD participants has been attached as Supporting Information I.

### 2.3. Design of Survey Tool, Quantitative Data Management and Analysis

A semistructured questionnaire was utilised for the quantitative survey. The questionnaire comprised of three sections: (a) participants’ demographics; (b) general farm characteristics; and (c) farmers’ production characteristics, costs and health practices. After the initial design, the questionnaire underwent a pretesting phase via administration to 10 fish farmers. Required edits were made, and a finalised version was approved (Supporting Information II). The questionnaire was administered to survey respondents through face‐to‐face interviews using the KoboToolBox application. Informed consent was obtained from the respondents prior to data collection, with an option to opt out or refuse any question during the interview process. The collected data were inputted into a Microsoft Excel 365 spreadsheet and cleaned. Statistical analyses were conducted using R Version 4.5.1 [21]. Descriptive analyses and logistic regression were performed using the stats package, and data visualisations were generated using ggplot2. Artificial intelligence (AI) was used to support the generation and verification of ggplot2 codes used in the R programming software. Descriptive statistics were used to summarise demographics and production characteristics, with results presented in tables and charts. Most production characteristics were obtained from the farmers during the questionnaire administration. However, the water area was calculated by multiplying the total number of ponds on the farm by pond size and converting the obtained figure to plots; 1 plot = 650 m^2^. Stocking density for grow‐outs was also calculated with the formula below^1^:

(1) Stocking density=Number of fish stocked nNumber∗size of grow−out pond per farm m2.

Costs of production were calculated in the following manner: In the questionnaire, respondents were asked to provide the amount they spent during the production cycle under several categories, including costs of rent, feed, stocking, diagnostic tests, and others. These were categorised into fixed and variable costs. They are defined thus: Fixed costs are not affected by the production scale. This included rent, pond maintenance and salary costs. Variable costs change depending on the production scale. This included feed, stocking, health management, fertiliser and electricity costs. Health management costs included the costs of medications, veterinary consultancy, water quality testing, biosecurity and laboratory diagnostic tests. Total costs were the sum of fixed and variable costs. Mortality costs included losses due to fish mortality only. All costs were converted from NGN to USD at the rate of USD 1 to NGN 1585 in accordance with average exchange rates in April 2025 [22]. Total costs were divided by the total weight of fish harvested during each production cycle to estimate the average cost of production per kg of fish harvested in each farm. Due to the non‐normal distribution of the data, costs are summarised as medians.

Inferential analyses were conducted using Pearson’s chi‐square, Fisher’s exact test, and logistic regression. Possible associations between production characteristics and disease outbreaks/survival rates, as well as production characteristics and health management expenses, were investigated. Disease outbreaks and health management expenses were coded as binary outcomes (0 = No, 1 = Yes), with ‘No’ as the reference category. Survival rate was categorised as < 50%, 50%–75% and > 75%, with < 50% used as the reference category. Each production characteristic was initially assessed for its association with the relevant outcome using Fisher’s exact test and Pearson’s chi‐square test. Variables with *p* < 0.20 were subsequently entered into the final multivariate model. A *p* < 0.20 threshold was used to retain potentially relevant variables that may not have reached the conventional p < 0.05 threshold utilised for the logistic regression model. Missing data were minimal and excluded from the relevant analyses. Regional‐level clustering was not explicitly modelled and farms were treated as independent observations. All 330 respondent farms were included in the multivariate analyses. All statistical tests were two‐sided, with an a priori significance level of *p* < 0.05 for multivariate analyses.

### 2.4. FGD, Qualitative Data Management and Analysis

A preliminary FGD was conducted prior to the questionnaire survey to provide contextual understanding and support the development of the questionnaire. Questions asked during this session included the most common NAHIs available and factors influencing farmers’ use of veterinary services. After reviewing results from the questionnaire, the research team developed another interview topic guide for a second FGD (Supporting Information III). This session was intended to provide further information regarding factors influencing the use of antimicrobials and NAHIs. Antimicrobials as defined here include medications developed for treating infectious diseases, including antibiotics, antivirals, and antifungals. Within this study, NAHIs are defined as all substances, strategies and tools farmers employed in preventing and treating diseases outside of conventional antimicrobials. This included herbs, antioxidants, multivitamins, probiotics and disinfectants such as salt and formalin [16].

The focus groups were conducted thus: Each session was moderated by two researchers, while a third researcher served as a note‐taker. The researchers had no prior relationships with most of the participants; however, some participating veterinarians were known to some members of the research team through professional interactions. Questions were asked sequentially, and participants were freely allowed to express their opinions. Responses were recorded via manual transcription and audio recordings; the latter were transcribed with TurboScribe and reviewed by the facilitators for accuracy.

Analysis was conducted based on Braun and Clarke [23]’s guide to reflexive thematic analysis. Two researchers independently read the transcripts multiple times to allow for data familiarisation. Visual maps were also generated. Initial codes were created independently by both researchers using the Taguette software. The researchers then met to discuss the codes, compare interpretations and refine the coding network. Differences in interpretation were resolved through iterative discussions and reflections. The researchers subsequently reviewed the final set of codes and sought potential themes. Final themes were decided after multiple rounds of discussion and reflection until both researchers were in agreement. Four candidate themes emerged regarding the disease management practices of farmers. A systems map was developed using LucidChart to graphically represent the interactions of the themes.

Data adequacy was assessed by comparing the findings across the two FGDs and considering whether the discussions generated new findings relevant to the study objectives. This assessment revealed that the discussions provided sufficient information power to triangulate the survey findings and identify consistent patterns across the two FGDs.

## 3. Results

### 3.1. Demographic Characteristics of Fish Farmers

The demographic characteristics of 330 study participants are summarised in Table 1. The majority of respondents (68.2%) were between the ages of 31 and 50. Most (85.8%) were male, and a significant proportion (71.8%) were tertiary educated. The majority (72.1%) had up to 10 years of farming experience.

**TABLE 1:** Demographic characteristics of study respondents.

Sociodemographics	Number (%)
Age (years)	
< 20	4 (1.21)
21–30	56 (16.97)
31–40	124 (37.58)
41–50	101 (30.61)
51–60	37 (11.21)
> 60	5 (1.52)
N/A	3 (0.91)
Gender	
Male	283 (85.76)
Female	47 (14.24)
State	
Kwara	173 (52.42)
Lagos	157 (47.58)
Level of education	
No formal education	2 (0.61)
Primary school	8 (2.42)
Secondary school	83 (25.15)
Tertiary education	237 (71.82)
Role on farm	
Owner	117 (35.45)
Owner and manager	92 (27.88)
Manager	65 (19.70)
Worker	56 (16.97)
Number of years in fish farming	
≤ 5	127 (38.48)
6–10	111 (33.64)
11–15	47 (14.24)
16–20	29 (8.79)
> 20	16 (4.85)

Abbreviation: N/A, not available.

### 3.2. Farm Characteristics and Production Practices

The majority of farms (69.7%) raised catfish alone, and the second‐largest proportion (23.6%) ran a mixed farming system that included catfish and tilapia (Figure 2). Most farms (75.2%) were small‐scale and had only up to 5 plots of water area (1 plot = 650 m^2^) (Table 2). The majority (70%) lacked a water reservoir. Farms were often in clusters, with a significant proportion (66.1%) sharing water sources with neighbouring farms. During the FGDs, farmers provided more context to this, observing that they often experienced disease outbreaks with other farms who were in close proximity to each other. An FGD participant stated: ‘*Most farms, because of the clusters […], the activities [taking place in] the [first farm] is going to affect [the second farm], [even though] physically you might not see the water seeping in [between farm ponds]’.—R09, veterinarian and catfish farmer.* Only 40% of farms relied at least partially on boreholes for their water supply; other farms used water from natural sources such as dams, rivers and springs (Figure 3). While 88.1% of farms kept records in general, only 27.6% kept health records, and even fewer (15.2%) kept records on water quality.

**FIGURE 2:**
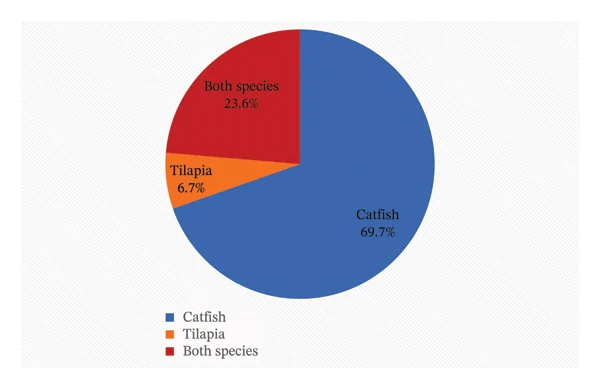
Species of fish raised on farms. The pie chart shows the proportion of the 330 surveyed farms reporting production of catfish, tilapia, or both (*n* = 330).

**TABLE 2:** Characteristics of fish farms.

Farm characteristics	Number (%)
Year of farm establishment	
≤ 2005	27 (8.18)
2006–2015	110 (33.33)
2016–2025	193 (58.48)
Total water area (plots; 1 plot = 650 m^2^)	
≤ 5	248 (75.15)
6–10	43 (13.03)
11–20	29 (8.79)
> 20	10 (3.03)
Reservoir water facilities	
None	231 (70.00)
≤ 2	61 (18.48)
3–5	26 (7.88)
> 5	12 (3.64)
Number of nursery facilities	
None	190 (57.58)
≤ 5	104 (31.52)
6–10	22 (6.67)
11–20	9 (2.73)
> 20	5 (1.52)
Use of shared water sources	
Yes	218 (66.06)
No	112 (33.94)
Record keeping	
Yes	291 (88.18)
No	39 (11.82)
Types of farm records kept (multiple‐choice)	
Feed	289 (87.58)
Production	244 (73.94)
Mortality	142 (43.03)
Health	91 (27.58)
Water quality	50 (15.15)

**FIGURE 3:**
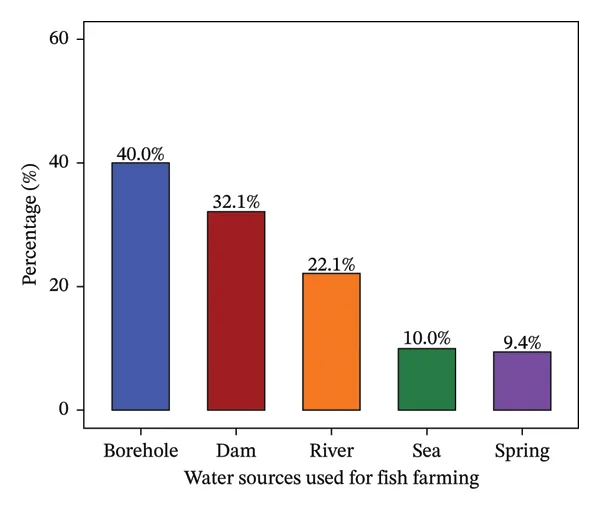
Water sources used on farms for aquaculture production. Bars represent the proportion of farms using each water source (*n* = 330, multiple‐choice).

Regarding production, 68.8% of farms stocked fish at the juvenile stage. Most farms (86.4%) sourced fish from private hatcheries rather than cheaper sources such as government hatcheries or from the wild (Table 3)**.** Most farms (87.9%) stocked more than 7 fish per m^2^ of water. More than 90% of farmers used commercial feeds. More than half of the farmers were not aware if these commercial feeds contained additives (vitamins, antibiotics, etc.). These findings were further explored in the FGDs, where a participant who worked as a feed miller stated that it was not usual practice to mix antibiotics with feed commercially: ‘*Generally, medicated feed is not something that is encouraged, because of several [reasons]. First, no antibiotics come cheap. Secondly, [why would you] treat [a fish] that is not sick?’—R10, feed miller/formulator.* Few farms (18.5%) added these substances to the feed by themselves prior to using them. Some farmers (32.1%) used pond additives, particularly salt, for disinfectant purposes.

**TABLE 3:** Production characteristics of farms.

Farm production system characteristics	Number (%)
Stage of fish stocked	
Juvenile	227 (68.79)
Fingerlings	78 (23.64)
Fry	12 (3.64)
Postfingerlings	7 (2.12)
Postjuvenile/grow out	6 (1.82)
Sources of fish stocked^∗^	
Privately‐owned hatchery	285 (86.36)
Farmer’s personal hatchery	55 (16.67)
Government hatchery	2 (0.61)
Others (wild)	3 (0.91)
Number of fish stocked per water area in the last production	
1000	104 (31.52)
1001–2000	85 (25.76)
2001–3000	47 (14.24)
3001–4000	31 (9.39)
> 4000	63 (19.09)
Stocking density (/m^2^)	
≤ 7	40 (12.12)
8–14	111 (33.64)
15–21	95 (28.79)
> 21	84 (25.45)
Sources of feed used	
Commercial	309 (93.64)
Home‐made (milled)	27 (8.18)
Aware if additives are used in purchased commercial feeds?	
Yes	154 (46.67)
No	176 (53.33)
Use of additives in feed (antibiotics, vitamins, etc.)	
Yes	61 (18.48)
No	269 (81.52)
Feed additives used (*n* = 61)^∗^	
Vitamins	20 (32.79)
Antibiotics	15 (24.59)
Amino acids	6 (9.84)
Herbs	3 (4.92)
Animal by‐products (offal, poultry waste)	2 (3.28)
Others (chemicals, probiotics and cooking materials)	23 (37.70)
Use of pond additives	
Yes	106 (32.12)
No	224 (67.88)
Pond additives used (*n* = 106)^∗^	
Salt	76 (71.70)
Formalin	15 (14.15)
Lime/mineral agents	9 (8.49)
Toxins (phosphide, chlorine, etc.)	6 (5.66)
Antibiotics	5 (4.72)
Others (organic waste, herbs and vitamins)	10 (9.43)
Total production cycle (months)	
1–3	24 (7.27)
4–6	161 (48.79)
7–9	136 (41.21)
> 9	9 (2.73)
Partial harvests per production cycle	
None	152 (46.06)
1–2	118 (35.76)
3–4	53 (16.06)
> 4	7 (2.12)
Farm yield per production cycle (kg/water area)	
≤ 500	78 (23.64)
501–1500	132 (40.00)
1501–3000	61 (18.48)
3001–4500	26 (7.88)
> 4500	33 (10.00)

^∗^Percentages might exceed 100% due to multiple responses.

Most farms (63.6%) yielded up to 1500 kg of fish per water area. The majority of farms (63.3%) had survival rates of > 75% in the last production cycle (Figure 4). Pearson’s chi‐square test identified some associations between production characteristics and survival rates (Supporting Information IV), but none of these were found to be independently associated in a logistic regression model (*p* > 0.99).

**FIGURE 4:**
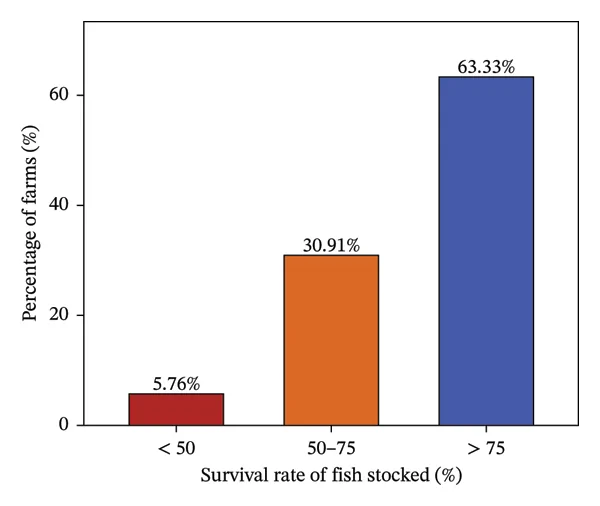
Survival rate of fish stocked on farms in the last production. Bars represent the proportion of farms within each survival category (*n* = 330).

### 3.3. Cost Expenditure of Farms

Farms spent a median of USD 1.20 per kg of fish harvested over the last production cycle (Table 4). Variable costs, including feed, stocking and health management, amounted to a median of USD 1.07. Feed accounted for a median of 91.3% of variable costs across all farms. Excluding feed, variable costs amounted to a median of USD 0.08 per kg of fish harvested.

**TABLE 4:** Production costs per production cycle in farms surveyed.

Cost per production cycle (/kg harvested)	Median (USD)	IQR (USD)
Total cost of production	1.20	0.93–1.74
Variable costs of production	1.07	0.85–1.40
Variable costs of production (excl. feed)	0.08	0.04–0.20
Health management costs	0.003	0.00–0.01
Medication costs	0.001	0.00–0.01

Health management accounted for less than 1% of variable costs. This included costs accrued from medications, water quality tests, consultancy fees, biosecurity and laboratory diagnostic tests. Biosecurity expenses included costs on pond netting and disinfectants. While 62.1% of farms spent on medication over the last cycle, only 20% and 15.5% of farms reported costs for biosecurity and water quality testing, respectively. Approximately 13% reported costs for consultancy and diagnostic fees. Figure 5 highlights the total proportion of health management costs allocated to the three most common categories. Generally, farmers spent 100% of their health management costs on medications and nothing on biosecurity or water quality. Among farmers who spent on the latter categories, tilapia farms had approximately three times the odds of spending on biosecurity (OR: 3.06, 95% CI: 1.38–6.81, *p* : 0.005) and water quality (OR: 3.29, 95% CI: 1.44–7.38, *p* : 0.004) than catfish farmers (Table 5). This was further supported by the FGDs, where tilapia farmers reinstated the need to keep optimum water quality because of the sensitive nature of tilapia: ‘*Tilapia are scaled fish, so they are more resistant [to infectious diseases]. The only issue is when you overstock and they have a lack of oxygen’.—R06, mixed (catfish and tilapia) farmer.* Farmers who used boreholes had eight times the odds of spending on water quality tests (OR: 8.07, 95% CI: 4.53–15.16, *p* < 0.001) and twice the odds of spending on biosecurity than farmers who used natural water sources only. Farmers who spent on biosecurity measures had about 71% lower odds of also spending on medications over the last production cycle than farmers who did not (OR: 0.29, 95% CI: 0.14–0.56, *p* : 0.001).

**FIGURE 5:**
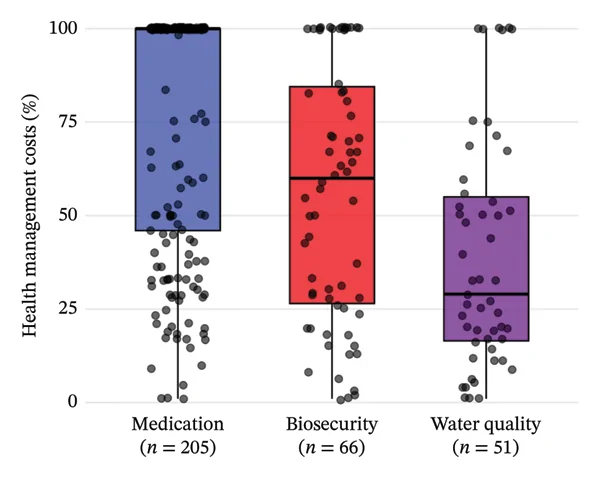
Proportion of health management costs apportioned in each farm to medication, biosecurity and water quality. The bars and lines represent the range of proportions, while the black lines represent the median proportion for each category. The (*n*) refers to the number of farms in the study that provided costs for specific categories. The black line on the bar represents how much the expenses in each category contributed to farms’ total health management costs for all farmers who spent in that category (median): 100% for medication, 60% for water quality, and 29% for biosecurity.

**TABLE 5:** Multivariate analysis (logistic regression) comparing spending on health management categories with selected farm characteristics.

Variables	Category	Medication		Biosecurity		Water quality	
		Odds ratio (95% CI)	*p* value	Odds ratio (95% CI)	*p* value	Odds ratio (95% CI)	*p* value
Type of fish raised	Catfish	*r*		*r*		*r*	
	Tilapia	1.42 (0.63–3.56)	0.415	**3.06 (1.38–6.81)**	**0.005**	**3.29 (1.44–7.38)**	**0.004**
	Mixed	0.99 (0.59–1.66)	0.971	0.80 (0.43–1.44)	0.470	1.15 (0.60–2.10)	0.66

Water sources used	Natural water sources	r					
	Borehole	**2.39 (1.54–3.79)**	**< 0.001**	**2.03 (1.27–3.26)**	**0.003**	**8.07 (4.53–15.16)**	**< 0.001**

*Note:* Bold values indicate statistical significance.

About 27% (*n* = 89) of survey respondents reported experiencing disease outbreaks in their farms in a cycle over the last year (Table 6). Disease outbreaks as described here refer to periods of prolonged sickness that affect most of the fish stock and cause significant and recurrent mortality, rather than random individual fish deaths. This distinction was further reflected in the FGDs, where one participant stated, ‘*One or two fish die from time to time. It is normal. I even [experienced this on my farm] last night. But I’ve not had any major outbreak since 2021’.—R04, catfish farmer.* Among farmers who experienced disease outbreaks, most farmers (47.2%) lost up to 10% of their stock, while 29.2% lost between 11% and 25% of their stock. There were no significant associations observed between disease outbreaks and farms’ production characteristics (*p* > 0.05). Out of 89 respondents who experienced disease outbreaks, 69 (77.5%) were able to provide sufficient information to estimate the monetary value of mortality‐related losses. Mortality losses were therefore calculated using the information provided by these farmers and are not representative of all farmers who experienced disease outbreaks. Among these farmers, the median mortality‐related loss was USD 0.047 per kg of fish harvested. Farmers who faced disease outbreaks spent approximately 1.7 times as much on health management as the general median (USD 0.005 vs. 0.003).

**TABLE 6:** Losses due to diseases and associated costs.

Disease losses	Values
Experienced a disease outbreak over the last year?	*n* (%)
Yes	89 (26.97)
No	241 (73.03)
Mortality losses due to diseases over the last year (*n* = 89)	n (%)
≤ 10	42 (47.19)
11–25	26 (29.21)
26–50	13 (14.61)
> 50	8 (8.99)
Disease and production costs (USD per kg harvested) (*n* = 69)	Median (IQR)
Mortality losses	0.047 (0.014–0.118)
Health management costs	0.005 (0.003–0.016)

### 3.4. Health Management Practices of Farmers

Surveyed farmers reported mostly diagnosing diseases on their farms by observation (71.5%) and experience (60.6%). Only 4.2% of farmers consulted private veterinarians for diagnosis. Farmers in the FGDs stated that they usually diagnosed bacterial, viral and fungal diseases through a combination of physical signs and lesions.

Most FGD participants stated they had experienced at least one disease outbreak during their time in the fishing business. Tilapia farmers tended to deal with more management‐related diseases, while catfish were considered more sensitive to infectious diseases. Among these, bacterial diseases were considered the most significant, as in this account: *Bacteria is more expensive [than other infections]. I can’t even count how many times I’ve experienced bacterial disease. I’ve lost numbers [of fish]. It’s something that is common. Every year, I have it.’—R08, Catfish farmer.* It should be noted that most farmers here based their diagnosis on their personal perceptions of what bacterial diseases looked like, rather than on confirmatory laboratory diagnosis.

Farmers in the FGD stated that they used antibiotics in managing diseases. Some farmers also used antibiotics for prevention. One farmer said: ‘*We use antibiotics for catfish, once we see that there might be any issues (change in appetite or behaviour). We use it to treat […]. But [as for] my neighbours, some of them use it for prevention [on their own farms]’.—R06, mixed (catfish and tilapia) farmer.* Antibiotics reported as being commonly used included tetracycline, penicillin, ciprofloxacin, doxycycline and chloramphenicol. A farmer described his use of antibiotics here: ‘*We use oxytetracycline 2 weeks after hatching if we notice there is mortality, then at 2-3 months in the grow-out pond we use aquaceryl plus [a neomycin and oxytetracycline mix] prophylactically’.—R05, mixed (catfish and tilapia) farmer.*

The NAHIs used by farmers as uncovered in the FGD included herbs, multivitamins, salt, charcoal, agricultural lime, and formalin. Relatively few farmers reported using probiotics and antioxidants. Herbs used included bitterleaf (*Vernonia amygdalina),* siam weed (*Chromolaena odorata*), moringa (*Moringa oleifera)*, hibiscus (*Hibiscus sabdariffa),* candle bush (*Senna alata)* and neem (*Azadirachta indica*). Farmers mostly used herbs by detaching leaves from the plant and adding them directly to the pond. Most NAHIs were used without ascribing to a treatment regimen and mostly for preventive purposes. One farmer said, ‘*I don’t have a withdrawal period for organic treatments. I can just [randomly] give the fish some [herbs] today. I can observe them tomorrow and give them moringa. Then, I can carry ewe Akintola (siam weed) and give them the next day. When I see [disease outbreaks], I can use salt and lime’.—R04, catfish farmer.*

Many farmers reported using a combination of antibiotics and NAHIs in a single outbreak. In this manner, antibiotics were often used secondary to NAHIs: ‘*When diseases happen […], I can place antioxidants or multivitamins in the pond. Then, if I see it persists, I will now treat [with antibiotics]. [Antibiotics are] the last resort’.—R09, veterinarian and catfish farmer.*

### 3.5. Factors Affecting Health Management Practices for Farmers

Themes uncovered from the FGDs centre on effectiveness, economics and awareness. Figure 6 summarises the interactions of factors identified and how they influence farmers’ health management decisions.

**FIGURE 6:**
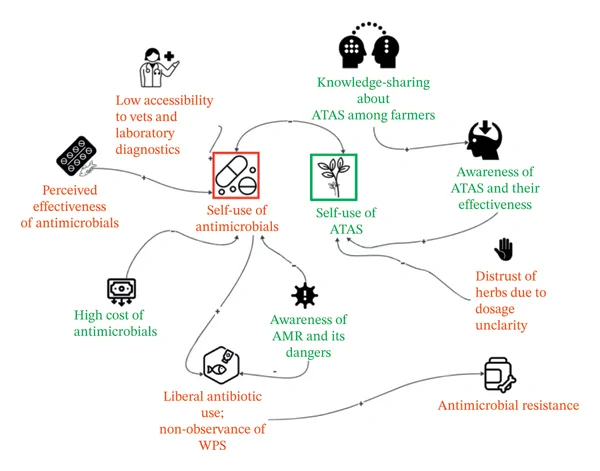
Systems map depicting the main factors affecting farmers’ use of antimicrobials and NAHIs, as well as possible contributing factors to AMR, based on farmers’ and veterinarians’ perspectives from the FGDs. WPs = withdrawal periods. Antimicrobials include antibiotics, antivirals and antifungals. NAHIs include herbs, probiotics, antioxidants and other factors mentioned earlier. Positive (+) arrows indicate a perceived positive influence of the preceding factor on the succeeding factor. Negative (−) arrows indicate a perceived negative influence. The arrows represent perceived influences, reported decision‐making pathways, or stakeholder‐described relationships, rather than actual causal effects.

#### 3.5.1. Perceived Effectiveness Drives Farmers’ Use of Antibiotics and NAHIs

Farmers stated that they chose treatment options based on their perspectives regarding effectiveness. Antibiotics were commonly used because farmers considered them the most effective treatment option. One farmer said:

“*Antibiotics work faster than herbs […] If we have outbreaks in fish, poultry or human beings, antibiotics work faster.”— R04, catfish farmer.*

Conversely, other farmers stated that they reduced their use of antimicrobials after noticing inefficacy. They attributed this to treatment failure due to AMR:

*“I’ve stopped [using antibiotics] for eight months now. I used it constantly [before], and there were no [positive] results. My vet always said it is resistance.”—R08, catfish farmer.*

According to farmers, effectiveness was prioritised over drug class or type. This led to farmers engaging in practices such as the use of poultry‐formulated antimicrobials for fish. One farmer stated:

*“We use Centerdicox [anticoccidial drug for poultry] as an antibiotic. We [use Centerdicox] because it works for us. We use it for any problem and add it to our fish feed.”—R03, mixed (catfish and tilapia) farmer.*

The NAHIs, particularly herbs, were also considered quite effective by many farmers, especially as long‐term prophylactic agents:

*“The herbs are very effective. If I thought they were not effective, I wouldn′t use them.”—R04, catfish farmer.*

However, other participants expressed scepticism about the efficacy of herbs due to a lack of dosage standardisation. A participant said:

*“ I’ve never used [herbs]. But I′ve seen plenty of farmers use [them]. They will tell you that it works. In my opinion, they are just lucky. How do you know which [dosage] concentration to use?”—R09, Veterinarian and catfish farmer.*

#### 3.5.2. High Dependence on Other Farmers’ Advice; Low Reliance on Veterinarians and Laboratory Diagnostics

Farmers often relied on their peers for guidance during disease outbreaks. This was a common means by which farmers learnt of, and adopted, the use of NAHIs in their fish. One farmer said:

“*We discuss our challenges among ourselves and give diagnosis based on our experiences. […] I advised someone to use bitterleaf and charcoal […], and it worked.”—R02, tilapia farmer.*

However, most farmers did not rely on veterinarians or laboratory diagnostics. Some farmers attributed this to a lack of awareness of their availability and due to distance. One farmer said:

“*We don’t consult anybody [due to] unavailability of laboratories.”—R05, mixed (catfish and tilapia farmer).*

Veterinarians in the group stated that most farmers contacted them for diagnosis only after their self‐prescribed antimicrobials had stopped being effective. A participant said:

*“When samples come to the lab, we run antimicrobial sensitivity testing and when we see the results, most of the pathogens are [already] resistant to the common antibiotics. The antibiotics [usually] found to be resistant are oxytetracycline, ampicillin, gentamicin […] but Levofloxacin is [usually] still potent.”— R13, veterinary laboratory scientist.*

Some farmers did not approach laboratories even after suspecting drug resistance. Instead, ‘tests’ were carried out on the farm to determine antimicrobial sensitivity, which also involved the use of more antimicrobials:

*“The test I do is on the farm. The moment I apply [an antibiotic], if I see it is not working, then [I conclude that] my fish is resistant. When there are no results, I will try another [antibiotic].”—R08, catfish farmer.*

#### 3.5.3. Cost‐Saving Measures Reduce Farmers’ AMU While Driving AMR Risk Practices

Farmers used antimicrobials as a second‐line treatment because of their high cost. One farmer said:

*“Before, I used to go for antibiotics, but when I discovered that the cost of buying it is more than that of the [herbs], I now go for [herbs] first.”— R16, catfish farmer.*

To preserve profit margins, farmers with an integrated fish‐farming system used their poultry waste (dead poultry, poultry faeces and dead fish) for food for fish. This was primarily practised for catfish, as tilapia were considered too sensitive for these. Participants also stated that antibiotics were heavily used in poultry practices. One FGD participant said:

*“Personally, I encourage [fish-chicken farming] a lot because waste from poultry is a feed to fish […] This is for catfish not tilapia. Tilapia is very sensitive. A little disturbance on tilapia and you will see mortality within 24 h. […] Broiler [chicken] is heavily laden with antibiotics.”—R10, feed miller/formulator.*

Farmers also tended to sell fish for human consumption after the fish had become resistant to all antimicrobials, without observing withdrawal periods. Veterinarians sometimes encouraged these practices, as in this account:

“*I had another client. All the fish were dying. We ran the tests, [all antibiotics] were resistant. I advised the farmer [to] just sell the fish […] I could not tell the farmer to condemn [and destroy the fish]. Do we have policies on ground for [governmental compensation]?”— R09, veterinarian and catfish farmer.*

It was also speculated that farmers would approach formal veterinary services more if they were rendered cheaper by government subsidies. A participant said:

*“If the government can subsidise the system for […] cost of diagnostics, this would improve the turnout of the farmers for lab diagnosis.”— R13, Veterinary laboratory scientist.*

#### 3.5.4. Farmers’ Awareness of AMR Led to a Reduction in AMU

Some farmers had learnt about AMR through aquaculture seminars, the internet, or large‐scale commercial vendors who rejected their products for having high antimicrobial residue content. One farmer recalled:

*“I was supplying one big farm in Ibadan. If you carry your bird to them, they will first […] test. If there is any sign of antibiotics, they will not collect the chicken from you.” R04, catfish farmer.*

According to farmers, this awareness about AMR caused either a switch to NAHIs, particularly herbs, or not attempting to resell their dying fish, as stated here:

*“ Aside from the [high cost of antibiotics], it has come to my knowledge that the [antibiotic] residues [in fish] come to humans. After a lot of research, I know [AMR] is causing many [problems] in humans. Now, I will rather dispose [my sick fish] than use antibiotics.”—R03, tilapia farmer.*

In addition, farmers reported that awareness of the importance of withdrawal periods also caused them to be judicious with their use of antimicrobials. This same effort was also exerted for NAHIs that necessitated withdrawal periods, like formalin:

*“That’s why I stopped using formalin. I’d rather not use formalin on my fish because of that withdrawal period. I must consider, what if it is my family that is buying that fish? So I stopped using it.”—R08, catfish farmer.*

## 4. Discussion

### 4.1. Demographic Characteristics of Farmers

Most respondents were male, had fewer than 10 years of farming experience and managed small‐scale operations. Similar findings have been reported in other studies conducted across Nigeria [24–26]. The gender distribution observed in this study aligns with global trends, which consistently indicate a predominance of males [26]. In Nigeria, this disparity occurs due to the interplay of cultural gender roles in aquaculture. Men are predominant in fish farming and rearing activities, while women are more involved in processing and marketing fish products [26–28]. The relatively short farming experiences among farmers here may reflect two factors identified from previous studies: first, commercial aquaculture is a relatively new venture in Nigeria; and second, fish farmers exit the industry at significant rates, mostly due to financial pressure from high production costs [26]. These factors cause high turnover rates and lead to the majority of aquaculture farmers being new entrants and less experienced. Okocha et al. [27] observed that farmers with up to 10 years of experience relied on antimicrobials for health management significantly more than farmers with more experience. Another notable finding is the high proportion of tertiary‐educated fish farmers in our survey, which aligns with other aquaculture studies but contrasts with Nigerian farmers in other agricultural sectors [27, 29]. This may be explained by the fact that aquaculture was initially introduced in Nigeria as an urban occupation, thereby attracting higher‐income earners with access to formal education [26].

### 4.2. Production Practices Influencing Disease Outbreaks

Farmers demonstrated optimal production practices such as record keeping, using salt as a pond disinfectant and relying on private industries for their production inputs, in alignment with findings from other Nigerian studies [26, 27, 30]. All of this likely contributed to the majority of surveyed farmers not reporting disease outbreaks over the last production cycle. However, FGD participants stated that they had experienced outbreaks at least once during the course of their farming business. These outbreaks might have been due to other risky practices observed, including water management [31]. Most farmers relied on natural water sources, and only a few kept records or allocated resources to water quality tests. In contrast with this, the World Organisation of Animal Health [32] recommends that fish farmers use treated water sources or disinfect natural water sources prior to use in fish farming [31]. Farmers who used boreholes (a cleaner water source than wells and dams) were more likely to apportion costs to water quality tests, suggesting that optimum water management practices were implemented by a minority of farmers. Farms also tended to share water sources due to the clustered nature of most farms. Cluster farming is a relatively common practice in Nigeria, as it allows farmers to reduce land costs while raising their fish within a supportive community [26]. However, FGD participants stated that they had observed that diseases were easily transmitted across cluster farms, likely due to run‐off. Other studies have also similarly identified that farm proximity and use of shared water sources are the highest risk factors for disease transmission across farms [33].

The stocking density on most farms surveyed was higher than the recommendations from existing literature (7 fish/m^2^ optimum for both species) [34, 35]. Other studies have observed that high stocking density affects fish health and productivity, leading to increased susceptibility to diseases. Overstocking has also been previously recorded to have a negative impact on water quality, as fish waste builds up more quickly in overstocked ponds, leading to a release of toxic gases and affecting fish health [34, 35]. Another production practice potentially contributing to disease outbreaks was the type of fish species reared. Catfish farming was more prevalent than tilapia, consistent with established trends in Nigerian aquaculture. This pattern has been associated with higher cultural preference [3, 26]. Participants stated that they observed catfish to be more susceptible to infectious diseases than tilapia. While this was based on participant observations, our findings here align with worldwide trends, as global antimicrobial consumption is estimated to be almost three times higher in catfish production than in tilapia [36]. Within our study, we also key differences in production practices for both species. Catfish farmers in the FGD stated they used poultry waste as feed, and surveyed catfish farmers were less likely to report spending on biosecurity and water management. Suboptimal production practices have been observed in other studies to significantly enhance catfish’s vulnerability to diseases [37]. Using organic materials as feed increases bacteria within ponds and subsequently the risk of pathogen transmission [38]. It is possible that these practices contributed to the high frequency of disease outbreaks FGD reported for catfish when compared to tilapia. However, it should be noted that our inferential analysis did not demonstrate an independent association when inferential statistics was conducted between production practices/characterisitics and survival rate or the occurrence of disease outbreaks over the last production cycle. Thus, while these factors have been identified in other studies as possible contributors to disease outbreaks, we cannot conclude from our quantitative findings that there were associations between these and disease occurrence. The non‐observance of association might be due to reasons of confounding and imbalance in group sizes for some production characteristics. Diseases are often multifactorial, and the absence of an observed association may reflect the interplay across multiple production factors. For instance, farmers with good water management may have overstocked their ponds, leading to no observable differences in disease incidence. In addition, while FGD participants stated that catfish were more susceptible to infectious diseases, the relatively fewer tilapia farms in this survey may have limited the ability to corroborate this via statistical analyses.

Most farmers surveyed reported spending on medication rather than prophylaxis (biosecurity and water quality testing) or veterinary care (consultancy and diagnostics). This implies a self‐managed and reactive approach to health management. Farmers who spent on biosecurity were less likely to purchase medications over the last production cycle. This may suggest they needed to use them less than farmers who did not spend on biosecurity. Previous studies have similarly established the importance of effective biosecurity and disease prevention in reducing disease outbreaks [17]. Farmers stand to reduce disease outbreaks and subsequently mortality by investing in biosecurity and water quality expenses at the outset of the production cycle [14, 15].

### 4.3. Use of NAHIs on Farms

Farmers used a variety of NAHIs in managing diseases on their farms, including herbs, antioxidants and materials like salt and formalin. Herbs identified in this study have previously been documented to possess efficacy against diseases through strengthening immunity, acting as disinfectants and exhibiting antimicrobial activity. Experimental studies have also attempted to identify effective dosages of these herbs [39, 40]. For example, tilapia fed with a fermented moringa leaf and seed mixture at a 10% concentration resisted the effects of *A. hydrophila* [40]*.* Likewise, catfish exposed to 4 mL/L leaf extract of Siam weed exhibited a 100% survival rate against *Staphylococcus aureus* [39]. However, there is still a lack of field trial studies, as well as little to no translation of current research findings into actionable solutions for farmers. Thus, there are no clearly defined recommended dosage formulations for use in aquaculture production.

In our study, a significant portion of farmers in this study appeared to use at least one NAHI in their production; however, there was a lack of cohesion regarding their use, as farmers could use them for disinfectant, prophylactic, or therapeutic purposes, or all three. As there were many NAHIs available, farmers could also rely specifically on a subcategory of NAHIs while not using the rest. For example, FGD participants reported being more aware of and using herbs than probiotics or antioxidants. This is similar to findings in another Nigerian study, where the majority of fish farmers were reported to have negligible information on probiotics [41]. Farmers in our study reported learning of NAHI use from neighbouring farmers rather than other information sources such as aquaculture seminars or information posters, also consistent with other studies [41]. While this allowed some farmers to engage in NAHI use, a lack of standardised information might have led to the arbitrary manner of use observed. Farmers’ awareness of NAHIs, and consequently adoption, can be significantly increased through the use of communication mediums of the internet, mass media, or training seminars. Here, farmers can be fully presented with a comprehensive overview of all available NAHIs within the Nigerian aquaculture sphere, particularly those which have been standardised (probiotics, antioxidants, etc.), where to procure them, and their potential applications.

Farmers in the FGDs regarded NAHIs as less effective than antibiotics, with farmers stating they adopted antibiotics for more critical outbreaks. On the contrary, NAHIs were used prophylactically and for the first stage of disease outbreaks. Existing laboratory research, however, has demonstrated that some NAHIs in use by farmers also have promising bactericidal and bacteriostatic properties [39, 40]. An issue raised by some farmers was a lack of clarity about dosage formulations. It is possible that farmers regard NAHIs as less effective because they were using dosages below the effective therapeutic dose. The mode of use by farmers may also reduce the effective concentration of the NAHIs. For instance, some farmers reported using herbs in their raw forms without any sort of additional processing. In contrast, laboratory trials assessing herbal efficacy involve processing procedures that include fermenting, drying and powdering [40]. While there are currently no defined dosage formulations for most of these NAHIs, presenting farmers with recent research evidence may also enable them to gain some perspective about what dosage concentrations and preparations are most likely to be effective.

### 4.4. AMU and Veterinary Consultations

Antimicrobial use among farmers was largely indiscriminate, including nonprescribed use and the use of poultry formulations in fish. The most commonly used antibiotic classes were β‐lactams and tetracyclines, which have been recorded to be widely employed for aquaculture production in Nigeria and worldwide [42, 43]. Farmers also reported the use of chloramphenicol, an antibiotic listed by Nigeria’s National Agency for Food and Drug Administration and Control [44], among banned veterinary drugs for use in food‐producing animals. Several other countries have also banned chloramphenicol due to the risk of human disease associated with its residues [44, 45]. Farmers in the FGDs noted that antibiotics did not seem to be as effective as before, corroborated by veterinarians’ reports of observing multiple resistance across fish farms after conducting laboratory testing. Similarly, other studies have reported high levels of antimicrobial residues in fish and fish products on Nigerian farms [10, 27].

Farmers tended to self‐prescribe due to their easy access to medications. Still, not all antimicrobials were easily accessible. Veterinarians in the FGDs stated that levofloxacin usually remained potent even when widespread resistance was observed. Levofloxacin is a fluoroquinolone antibiotic and has been classified as a Highest Priority Critically Important Antimicrobial (HP‐CIA) by the World Health Organisation [46], with its use heavily regulated in veterinary medicine [47]. Findings in our study indicate that farmers did not typically self‐prescribe levofloxacin, suggesting limited access to this antibiotic. It is likely that the distribution pathways of levofloxacin in Nigeria are stricter than for accessible antibiotics like tetracycline, with possible supply chain gaps that allow farmers easy procurement of certain medications.

Farmers stated they reduced their AMU following an increase in awareness about AMR risks. Awareness has been recommended by the Food and Agriculture Organization as an effective tool for promoting antimicrobial stewardship within the aquaculture industry [39]. Farmers also stated they reduced AMU after their products were rejected by vendors for exceeding the antibiotic maximum residue limits (MRLs). According to FAO, rejection of fish products within export markets has led to several countries implementing stricter regulations [48]. It is debatable whether these rejections can trigger appreciable impacts in Nigeria, as many small‐scale farmers sell directly to final consumers, bypassing residue tests required by export laws and larger vendors [49]. Thus, fish previously rejected by vendors may still enter the market through this unregulated channel, creating a major risk pathway for the transmission of ARGs to humans [50].

Presently, AMU regulations in Nigeria restrict the use of antimicrobials to licensed veterinary personnel only. This law did not constrain farmers′ reported use, reflecting weak enforcement [51]. Farmers also stated that they put diseased fish up for sale without observing withdrawal periods or MRLs. Veterinarians tacitly encouraged these practices, justifying them based on governmental noncompensation. It must be noted that even in countries where AMU laws are enforced, farmers are not usually compensated when their products are destroyed due to residue violations. Rather, farmers are expected to comply with laws regarding WPs and MRLs in order to reduce the possibility of their products being condemned without compensation [52, 53].

Less than 5% of farmers consulted veterinarians, and only 13% reported diagnostic and consultancy costs as part of their health management expenses over the last cycle. This aligns with findings from Khor and Bodunde [37], where only 1.34% of Nigerian fish farmers relied on veterinarians for health management. Veterinarians reported being consulted only after the most common antimicrobials had been found to beineffective, primarily due to reasons of unavailability and cost. In a Hong Kong study, a subsidised veterinary scheme was implemented for fish farmers through a partnership between the government and higher educational institutions. This led to a higher dependence of farmers on veterinary services while reducing the prevalence of AMR in fish products due to commonly misused antibiotics [54]. Likewise, the introduction of similar programmes in Nigeria may lead to a decline in AMR‐risky practices such as on‐farm sensitivity ‘testing’ and the use of antimicrobials without laboratory diagnostic tests.

### 4.5. Study Limitations

Farm clustering was not accounted for in the analysis, therefore raising the potential of clustering bias. Given that a substantial proportion of farms shared water sources and operated within farm clusters, the observed associations from the quantitative analyses should be interpreted with caution, as the lack of adjustment for clustering may have affected the estimated associations and their statistical significance. Most of the data obtained regarding production characteristics, disease prevalence, and cost expenditure were not directly measured but recorded from participants’ memories. While we stressed the need for honest reporting throughout data collection, there is a risk of recall bias and social desirability bias. This may have been particularly relevant to production costs and AMU, as participants may have been less likely to disclose this due to concerns about how their practices might be perceived or context‐specific ideas of keeping production costs as private information. However, the use of a structured questionnaire allowed us to capture participants’ perspectives, which is particularly relevant to a study examining how and why farmers managed their farms and used antimicrobials and NAHIs. In addition, the inclusion of two focus groups allowed participants to discuss their experiences and practices openly.

## 5. Conclusions and Recommendations

Our study presents important insights into health management practices and factors influencing antimicrobial and NAHI use. Our findings indicate a primary reliance on treatment strategies over prevention. Farmers report their NAHI use as being influenced by awareness and affordability, but perceptions about their lack of efficacy for more critical outbreaks caused a heavy dependence on antimicrobials. Based on our findings, we recommend field trials to confirm effective dosage formulations for available NAHIs, particularly for those with regulatory and standardised formulations. Similarly, more laboratory and clinical trials could be conducted on widely used herbs to determine their efficacy. Value chain analysis can help map out distribution pathways for commonly used antibiotics, identifying probable points of actions that may be targeted to reduce farmers’ access to these drugs. Aquaculture seminars organised by the government and higher educational institutions can provide farmers with evidence‐based recommendations on optimal management practices and NAHIs. Antimicrobial regulatory laws should be publicised across fishing communities, with enforcement and penalty standards set. Veterinary services can be increasingly made available through subsidies and partnerships with higher institutions.

## Author Contributions

Ismail Ayoade Odetokun: conceptualisation, funding acquisition, methodology, investigation, project administration, resources, writing–review and editing, supervision. Jadesola Juliana Ajao: writing–original draft, writing–review and editing, investigation, validation, formal analysis, visualisation, data curation. Afisu Basiru: investigation, data curation, formal analysis, methodology, writing–original draft, writing–review and editing. Rodiat Olabisi Omotoso: investigation, data curation, writing–original draft, writing–review and editing, formal analysis, validation. Mutiat Adenike Adetona: investigation, writing–original draft, formal analysis, data curation. Wasiu Adeyemi Jimoh: investigation, data curation, writing–original draft. Somrat Adeola Abdulsalami: investigation, data curation, writing–original draft, writing–review and editing. Abdulfatai Aremu: investigation, data curation, writing–original draft. Mutiat Olaitan Mohammed: investigation, data curation, validation, writing–original draft. Samira Sarih: investigation, writing–review and editing. Enric Gisbert: writing–review and editing. Maria Dolores Furones Nozal: writing–review and editing. Elena Garcia Fruitós: conceptualisation, investigation, writing–review and editing. Anna Aris: conceptualisation, investigation, funding acquisition, writing–review and editing. Silvia Torrecillas: conceptualisation, investigation, writing–review and editing. Mahmoud Eltholth: conceptualisation, investigation, funding acquisition, methodology, project administration, supervision, writing–review and editing.

## Funding

This work was carried out with financial support from the International Development Research Centre (IDRC), Canada, and the Global AMR Innovation Fund (GAMRIF), part of the U.K. Government’s Department of Health and Social Care (DHSC) (grant number: 110337). These finances supported data collection, analysis, interpretation, report writing and publication drafting.

## Disclosure

All authors have read and approved the final version of the manuscript. The corresponding author, Mahmoud Eltholth (mahmoud.eltholth@rhul.ac.uk), had full access to all of the data in this study and takes complete responsibility for the integrity of the data and the accuracy of the data analysis and affirms that this manuscript is an honest, accurate and transparent account of the study being reported. No important aspects of the study have been omitted, and any discrepancies from the study as planned have been explained. The funding bodies had no involvement in the study design, collection, analysis, or interpretation of data.

## Ethics Statement

All study protocols were approved by the University of Ilorin Research Ethical Review Committee (approval number: UERC/ASN/2024/2915) and the Royal Holloway University of London (Application ID 4210). The data collection process was conducted in accordance with the Declaration of Helsinki [55]. Verbal and written consent were obtained from all survey and FGD participants. Confidentiality assurances were also communicated, with a clear option to opt out at any time during survey data collection and FGDs.

## Conflicts of Interest

The authors declare no conflicts of interest.

## Endnotes

^1^While only some farms stocked fry, all farms had a grow‐out pond, which was used for calculations.

## Supporting Information

Additional supporting information can be found online in the Supporting Information section.

## Supporting information


**Supporting Information 1** Supporting Information I. List of FGD participants.docx: demographics and IDs of FGD participants.

**Supporting Information 2** Supporting Information II. Questionnaire survey.docx: questionnaire survey.

**Supporting Information 3** Supporting Information III. Interview guide.docx: interview guide for focus group discussion.

**Supporting Information 4** Supporting Information IV. Bivariate analysis.docx: Pearson’s chi‐square test—association between survival rate and demographics, farm characteristics.

## Data Availability

The data that support the findings of this study are obtainable from the corresponding author upon reasonable request.
