# Supplementary material for: Health Management Practices, Antimicrobial Use and the Adoption of Alternatives Among Nigerian Fish Farmers: A Mixed‐Methods Study
Source: Vet Med Int. 2026 Oct 28;2026:3100926. doi: 10.1155/vmi/3100926 (PMC13717229; doi:10.1155/vmi/3100926)
Supplement: Supplementary file 1 [file VMI-2026-3100926-s001.docx]

**Supplementary Information I - IDs and Demographics of Focus Group Discussion Participants**

| S/N | Participant ID | SEX | Farm Size | State | Job Roles |
| --- | --- | --- | --- | --- | --- |
| 1. | FGD001 | M |  | Kwara | Catfish farmer |
| 2. | FGD 002 | F |  | Kwara | Breeder and catfish farmer |
| 3. | FGD 003 | M |  | Kwara | Breeder and mixed (catfish and tilapia) farmer |
| 4. | FGD 004 | M |  | Kwara | Catfish farmer |
| 5. | FGD 005 | M |  | Lagos | Mixed (catfish and tilapia fish farmer) |
| 6. | FGD 006 | M |  | Kwara | Mixed (catfish and tilapia fish farmer) |
| 7. | FGD 007 | M |  | Kwara | Mixed (catfish and tilapia) farmer |
| 8. | FGD 008 | M |  | Kwara | Catfish farmer |
| 9. | FGD 009 | F |  | Kwara | Veterinarian and catfish farmer |
| 10. | FGD 010 | M |  | Kwara | Feed miller/formulator |
| 11. | FGD 011 | M |  | Kwara | Catfish farmer |
| 12. | FGD 012 | M |  | Kwara | Veterinarian and catfish farmer |
| 13. | FGD 013 | M |  | Lagos | Veterinarian and laboratory scientist |
| 14. | FGD 014 | M |  | Kwara | Tilapia farmer |
| 15. | FGD 015 | M |  | Kwara | Mixed (catfish and tilapia farmer) |
| 16. | FGD 016 | M |  | Kwara | Catfish farmer |
| 17 | FGD 017 | M |  | Lagos | Mixed (catfish and tilapia farmer) |
|  |  |  |  |  |  |
|  |  |  |  |  |  |
|  |  |  |  |  |  |
