# Supplementary material for: Health Management Practices, Antimicrobial Use and the Adoption of Alternatives Among Nigerian Fish Farmers: A Mixed‐Methods Study
Source: Vet Med Int. 2026 Oct 28;2026:3100926. doi: 10.1155/vmi/3100926 (PMC13717229; doi:10.1155/vmi/3100926)
Supplement: Supplementary file 2 [file VMI-2026-3100926-s002.docx]

**Health management practices, antimicrobial use and the adoption of alternatives among Nigerian fish farmers: a mixed-methods study**

Corresponding author: Mahmoud Eltholth, [mahmoud.eltholth@rhul.ac.uk](mailto:mahmoud.eltholth@rhul.ac.uk)

Department of Health Studies, Royal Holloway University of London, Egham TW20 0EX, UK.

# Supplementary Information II - Questionnaire Survey.

**Section 0: QS details**

| Survey code: | __/_ _/_ _ _/_ _ _ | (State/Village/Farm Sn) | |
| --- | --- | --- | --- |
| Interviewer’s name: |  |  |  |
| Interview date: | (DD/MM/YEAR) | | |
| Consent obtained: | 1=Yes 2=No | | |

**Section 1: Interviewee details**

| Interviewee’s age: | /Years |
| --- | --- |
| Interviewee’s gender: | 1=Male 2=Female 3=Others 4=Prefer not to say |
| Level of education: | 1= Illiterate,  2= Literate, no formal education,  3= Primary school,  4= Secondary School  5= College/Polytechnic/University  6= Post graduate  7= Other: Specify_________________________________________) |
| Role in the farm | 1 = Owner 2 = Manager 3 = Owner and manager  4 = Worker 5 = Tenant 6 = Other: (Specify_____) |
| No. of years in aquaculture? /Years | |

**Section 2: Farm characteristics**

1. When was this farm constructed? _____________________/(year of construction)
2. What is the total land area of this farm (*including everything*)? _____________/Acre
3. What is the total water area of this farm including all ponds? __________/ Acre
4. For this farm, please complete the following table.

| Type of the water facility | Number | Total area/Acre |
| --- | --- | --- |
|  |  |  |
| Reservoir |  |  |
| Dedicated nursery |  |  |
| Culture\grow out |  |  |
| Water treatment pond |  |  |
| Other (Specify____) |  |  |

5. What is the source(s) of water for this farm? *(Could be more than one source)*

1= Water directly from sea	2=Borehole water	3=Tidal creek

4=River	5=Spring	6=Others

6. Any other users sharing the same water source before or after you?

1=Yes	2= No	99=Don’t know

7. Do you/someone else keep records for this farm?

1=Yes, 2= No, 99= Don’t know

8. If Yes, How do you keep records? *(Can be multiple answers)*

1= In book	2= On paper 3= On computer 4= On mobile phone 5= On wall chart	6= Other

9. Which areas do this farm have records for?

| Record | 1=Yes, 2= No, 99= Don’t know |
| --- | --- |
| Feed |  |
| Water quality |  |
| Chemicals and health products (treatment) |  |
| Treatment losses/mortality |  |
| Farm outputs |  |

**Section 3: Production, health and disease management practices.**

10. What were the production characteristics for the last completed production cycle?

|  | Last completed cycle |
| --- | --- |
| Stocking date | DD/MM/YY |
| Number of frys stocked |  |
| Stocking density/m^2^ |  |
| Age of frys at stocking |  |
| Number of days from stocking to final harvest |  |
| Survival rate (%) |  |
| Date of final harvest | DD/MM/YY |
| Number of partial harvests did you perform/cycle |  |
| The total yield (Kg)/cycle |  |

11. Over the last production cycle, what was the source of frys?

1=Own hatchery	2=Government hatchery	3=Private hatchery

4=Wild sources	5=Others (Specify_______________________________________)

12. What type of feed do you use for this farm?
13. What is the source of feed?
14. For commercial feed do you know if there are any feed additives?
15. Do you use any feed additives to the feed before giving to the fish?

1=Yes	2= No	99=Don’t know

16. If yes, what do you use?

| Feed additives | Amount/unit | Reason |
| --- | --- | --- |
|  |  |  |
|  |  |  |
|  |  |  |
|  |  |  |
|  |  |  |
|  |  |  |
|  |  |  |

17. Do you use any additives to the pond water?

1=Yes	2= No	99=Don’t know

18. If yes, what do you use?

| Water additives | Amount/unit | Reason |
| --- | --- | --- |
|  |  |  |
|  |  |  |
|  |  |  |
|  |  |  |
|  |  |  |
|  |  |  |
|  |  |  |

19. Which diseases of Feddan do you know? *(Please let the respondent to list them out)*

………………………………………………………………………………………………………………………………………………………………………………………………………………………………………………………………………………………………………

20. How do you know you have a disease on your farm? *(Allow multiple)*

1=Experience	2=Other farmers experience	3=Fish behaviour / observation

4=Mortality rate	5=Private consultant	6=Government official

7=Others (Specify__________________________________________________________)

21. Over the last year, did you have disease problems on your farm? *(Please let the respondent list all disease problems then ask for the details one by one)*

| Disease problem description | How did you know? | What did you do? |
| --- | --- | --- |
|  |  |  |
|  |  |  |
|  |  |  |
|  |  |  |
|  |  |  |
|  |  |  |
|  |  |  |
|  |  |  |

22. For the disease problems you had last year, please complete the following table.

| Description of the problem including symptoms | Approximate losses/mortalities % | Approximate cost of losses (NGN) |
| --- | --- | --- |
|  |  |  |
|  |  |  |
|  |  |  |
|  |  |  |
|  |  |  |
|  |  |  |
|  |  |  |
|  |  |  |

23. Over the last 5 years, did you attend training for any of the following *(details of training mentioned above)*?

| Training | 1=Yes 2=No | Training provider (1=Feed company, 2=Fish processing company 3=Government organisation 4= Private laboratory 5=Pharmaceutical company 6=Others ______) |
| --- | --- | --- |
| Pond management |  |  |
| Water quality management |  |  |
| Feed management |  |  |
| Disease and health management |  |  |
| Seed quality |  |  |
| Use of health products |  |  |
| Other (Specify____) |  |  |

24. Over the last production cycle, how much did the following production inputs cost you?

| Production inputs | Value/NGN |
| --- | --- |
| Lease/rent/production cycle, even if you own the land what is the value of rent for this farm/summer crop? |  |
| Pond maintenance |  |
| Frys/fingerlings |  |
| Feed |  |
| Total cost of labour |  |
| Electricity |  |
| Fuel including diesel/petrol |  |
| Fertilizers |  |
| Testing for water quality |  |
| Testing for Feddan diseases |  |
| Consultancy fee |  |
| Treatments/medications |  |
| Cost of staff training |  |
| Costs for biosecurity |  |
| Others (Specify) |  |

Thank you for your time and cooperation
