# Supplementary material for: Health Management Practices, Antimicrobial Use and the Adoption of Alternatives Among Nigerian Fish Farmers: A Mixed‐Methods Study
Source: Vet Med Int. 2026 Oct 28;2026:3100926. doi: 10.1155/vmi/3100926 (PMC13717229; doi:10.1155/vmi/3100926)
Supplement: Supplementary file 3 [file VMI-2026-3100926-s003.docx]

**Supplementary Information III - Topic Guide for Focus Group Discussion**

Corresponding author: Mahmoud Eltholth, [mahmoud.eltholth@rhul.ac.uk](mailto:mahmoud.eltholth@rhul.ac.uk)

Department of Health Studies, Royal Holloway University of London, Egham TW20 0EX, UK

BACA4RUMA PROJECT TEAM

UNIVERSITY OF ILORIN, NIGERIA

22ND OCTOBER, 2025

1. Can you describe the production chain of fish?

- What challenges do you face at each point of the production chain?
- What are the critical points where challenges of the production chain occur?
- What do you do to solve these challenges?

2. What is the average body weight of your fish at harvest?

- What are the maximum and minimum body weights?
- Do you know the body weight by just looking, or do you measure it with scales?

3. Do you think water quality is related to disease in fish?

- If yes, give reasons.

4. What are the main causes of diseases in your farms?

- Which challenges cost you the most money?
- Which challenge can you manage effectively, and which one do you still need training to manage?
- Which challenges do you feel you can solve easily, and which ones do you feel are hard to solve (no solution to treat, too expensive, others)?

5. How do you manage these challenges?

- How do you prevent these diseases?
- How do you treat them?
- How do your neighbours prevent/treat the diseases?

6. What are the best ways to manage these diseases?

- On your farm? On your neighbour’s farms?
- Are you confident in managing these diseases?
- What are the best ways your neighbours manage/treat these diseases

7. Do you use antibiotics?

- How frequently?
- How about your neighbours’ farms?
- Why do you or your neighbours use them?
- How do antibiotics affect your business, positively or negatively?
- How do antibiotics affect the industry or environment in your opinion?

8. Do you use alternatives to antibiotics?

- How frequently? How about your neighbours’ farms?
- Why do you or your neighbours use them?
- Do you prefer them to regular antibiotics?

9. Do you also farm chickens?

- Do you farm them near your ponds?
- Do you use antibiotics for them?
- Do you use any other treatments for chicken diseases?
- Are there other chicken farmers near your pond site?
- Do they use antibiotics or other treatments?

10. What do you feel about farming chickens and fish together?

- Benefits of farming them together? Disadvantages?
- Do you think antibiotics used in chicken can affect fish and vice-versa?
