# Supplementary material for: Health Management Practices, Antimicrobial Use and the Adoption of Alternatives Among Nigerian Fish Farmers: A Mixed‐Methods Study
Source: Vet Med Int. 2026 Oct 28;2026:3100926. doi: 10.1155/vmi/3100926 (PMC13717229; doi:10.1155/vmi/3100926)
Supplement: Supplementary file 4 [file VMI-2026-3100926-s004.docx]

**Health management practices, antimicrobial use and the adoption of alternatives among Nigerian fish farmers: a mixed-methods study**

Corresponding author: Mahmoud Eltholth, [mahmoud.eltholth@rhul.ac.uk](mailto:mahmoud.eltholth@rhul.ac.uk)

Department of Health Studies, Royal Holloway University of London, Egham TW20 0EX, UK

**Supplementary Information IV: Pearson’s chi-square test - Association Between Survival Rate and Demographics, Farm Characteristics.**

| **Factors** | **Survival Rate (%)** | | | **Chi-square** | **Fisher’s Exact P-value** |
| --- | --- | --- | --- | --- | --- |
|  | <50 | 50 - 75 | >75 |  |  |
| **Age (years)** |  | | | 11.33 | .33 |
| <20 | 0 (0.00) | 2 (50.00) | 2 (50.00) |  |  |
| 21–30 | 2 (3.57) | 24 (42.86) | 30 (53.57) |  |  |
| 31–40 | 6 (4.84) | 40 (32.26) | 78 (62.90) |  |  |
| 41–50 | 8 (7.92) | 21 (20.79) | 72 (71.29) |  |  |
| 51–60 | 3 (8.11) | 12 (32.43) | 22 (59.46) |  |  |
| >60 | 0 (0.00) | 1 (20.00) | 4 (80.00) |  |  |
| **Gender** |  | | | 1.85 | .50 |
| Female | 1 (2.13) | 13 (27.66) | 33 (70.21) |  |  |
| Male | 18 (6.36) | 89 (31.45) | 176 (62.19) |  |  |
| **Level of Education** |  | | | 3.18 | .79 |
| No formal education | 0 (0.00) | 1 (50.00) | 1 (50.00) |  |  |
| Primary school | 1 (12.50) | 4 (50.00) | 3 (37.50) |  |  |
| Secondary school | 4 (4.82) | 24 (28.92) | 55 (66.27) |  |  |
| Tertiary education | 14 (5.91) | 73 (30.80) | 150 (63.29) |  |  |
| **Type of fish raised** |  | | | 12.67 | **.01*** |
| Catfish | 7 (3.02) | 78 (33.62) | 147 (63.36) |  |  |
| Tilapia | 2 (9.09) | 5 (22.73) | 15 (68.18) |  |  |
| Mixed | 10 (13.16) | 19 (25.00) | 47 (61.84) |  |  |
| **Year of farm establishment** |  | | | 10.13 | .43 |
| ≤ 2000 | 1 (12.50) | 2 (25.00) | 5 (62.50) |  |  |
| 2001–2005 | 2 (10.53) | 2 (10.53) | 15 (78.94) |  |  |
| 2006–2010 | 3 (8.57) | 8 (22.86) | 24 (68.57) |  |  |
| 2011–2015 | 6 (8.00) | 25 (33.33) | 44 (58.67) |  |  |
| 2016–2020 | 4 (2.94) | 44 (32.35) | 88 (64.71) |  |  |
| 2021–2025 | 3 (5.26) | 21 (36.84) | 33 (57.89) |  |  |
| **Water area (plots)** |  | | | 26.54 | **.00*** |
| ≤ 5 | 10 (4.03) | 86 (34.68) | 152 (61.29) |  |  |
| 6–10 | 1 (2.32) | 9 (20.93) | 33 (76.74) |  |  |
| 11–20 | 7 (24.14) | 6 (20.69) | 16 (55.17) |  |  |
| >20 | 1 (10.00) | 1 (10.00) | 8 (80.00) |  |  |
| **Number of dedicated nurseries** |  | | | 3.97 | .93 |
| None | 11 (5.79) | 60 (31.58) | 119 (62.63) |  |  |
| ≤ 5 | 7 (6.73) | 33 (31.73) | 64 (61.54) |  |  |
| 6–10 | 1 (4.54) | 6 (27.27) | 15 (68.18) |  |  |
| 11–20 | 0 (0.00) | 3 (33.33) | 6 (66.67) |  |  |
| >20 | 0 (0.00) | 0 (0.00) | 5 (100.00) |  |  |
| **Stage of fish stocked** |  | | | 17.14 | **.06*** |
| Fingerlings | 6 (1.28) | 35 (44.87) | 37 (47.44) |  |  |
| Frys | 0 (0.00) | 6 (50.00) | 6 (50.00) |  |  |
| Grow out | 0 (0.00) | 0 (0.00) | 3 (100.00) |  |  |
| Juvenile | 13 (5.73) | 59 (25.99) | 155 (68.28) |  |  |
| Post fingerlings | 0 (0.00) | 1 (14.29) | 6 (85.71) |  |  |
| Post juvenile | 0 (0.00) | 1 (33.33) | 2 (66.67) |  |  |
| **Number of fish stocked (n/water area)** |  | | | 15.89 | **.04*** |
| ≤1000 | 9 (8.65) | 25 (24.04) | 70 (67.31) |  |  |
| 1001 - 2000 | 1 (1.18) | 29 (34.12) | 55 (64.71) |  |  |
| 2001 - 3000 | 1 (2.13) | 13 (27.66) | 33 (70.21) |  |  |
| 3001 – 4000 | 4 (12.90) | 8 (25.81) | 19 (61.29) |  |  |
| >4000 | 4 (6.35) | 27 (42.86) | 32 (50.79) |  |  |
| **Total production cycle (months)** |  | | | 7.36 | .29 |
| 1–3 | 3 (12.50) | 8 (33.33) | 13 (54.17) |  |  |
| 4–6 | 8 (4.97) | 43 (26.71) | 110 (68.32) |  |  |
| 7–9 | 8 (5.88) | 46 (33.82) | 82 (60.29) |  |  |
| >9 | 0 (0.00) | 5 (55.56) | 4 (44.44) |  |  |
| **Number of partial harvests** |  | | | 9.33 | **.16*** |
| None | 12 (7.90) | 51 (33.55) | 89 (58.55) |  |  |
| 1 – 2 | 4 (3.39) | 28 (23.73) | 86 (72.88) |  |  |
| 3–4 | 3 (5.66) | 21 (39.62) | 29 (54.72) |  |  |
| >4 | 0 (0.00) | 2 (28.57) | 5 (71.43) |  |  |
| **Feed additives** |  | | | 9.58 | **.05*** |
| No | 11 (4.09) | 79 (30.27) | 172 (65.90) |  |  |
| Yes | 8 (13.11) | 20 (32.79) | 33 (54.10) |  |  |
| I don’t know | 1 (12.50) | 3 (37.50) | 4 (50.00) |  |  |
| **Pond additives** |  | | | 22.39 | **0.00** |
| No | 13 (6.07) | 56(26.17) | 155 (69.20) |  |  |
| Yes | 6 (5.66) | 54 (43.40) | 46 (50.94) |  |  |
| **Stocking density** |  | | | 2.12 | .91 |
| <8 | 3 (7.50) | 11 (27.50) | 26 (65.00) |  |  |
| 8 - 14 | 7 (6.31) | 30 (27.03) | 74 (66.67) |  |  |
| 15 - 21 | 5 (5.26) | 32 (33.68) | 58 (61.05) |  |  |
| >21 | 4 (4.76) | 29 (34.52) | 51 (60.71) |  |  |

*statistically significant at the p<.20 level.
